# Predictive association of *ABCB1* C3435T genetic polymorphism with the efficacy or safety of lopinavir and ritonavir in coronavirus disease-2019 patients

**DOI:** 10.2217/pgs-2020-0096

**Published:** 2021-03-24

**Authors:** Mohitosh Biswas

**Affiliations:** ^1^Department of Pharmacy, University of Rajshahi, Rajshahi-6205, Bangladesh

**Keywords:** *ABCB1* genetic polymorphism, COVID-19, lopinavir/ritonavir, P-glycoprotein, safety and efficacy

## Abstract

Lopinavir and ritonavir are substrates of permeability glycoprotein encoded by *ABCB1.* The efficacy and safety of these drugs is unknown in coronavirus disease-2019 (COVID-19) patients affected by *ABCB1* genetic variability. Patients carrying one or two copies of the *ABCB1* C3435T were predictively considered as risk phenotypes. It was predicted that risk phenotypes due to carrying either one or two copies of *ABCB1* C3435T were highly prevalent in Europe (76.8%; 95% CI: 75–78), followed by America (67%; 95% CI: 65–69), Asia (63.5%; 95% CI: 62–65) and Africa (41.4%; 95% CI: 37–46), respectively. It is hypothesized that a considerable proportion of COVID-19 patients treated with lopinavir/ritonavir inheriting *ABCB1* C3435T genetic polymorphism may be predisposed to either therapeutic failure or toxicity.

Until specific antiviral drugs have been developed and approved for the treatment of coronavirus disease-2019 (COVID-19), some drugs such as favipiravir, lopinavir (LPV)/ritonavir (RTV), ribavirin, hydroxychloroquine/chloroquine and remdesivir have been recommended for the management of infection caused by SARS coronavirus-2 (SARS-CoV-2) as described elsewhere [1–3]. It has been found that a considerable proportion of COVID-19 patients infected with SARS-CoV-2 in likely clinical conditions were being treated with a combination therapy of LPV/RTV as first-line antiviral therapy [4]. However, there is wide variability in LPV/RTV response in COVID-19 patients as evidenced in clinical studies; randomized clinical trials of LPV/RTV have failed to prove superior efficacy compared with standard therapy [5,6]. Although many factors may trigger the clinical outcomes associated with using LPV/RTV, one of these may be genetic factor provoking drug response variability in addition to adverse clinical outcomes.

Being a substrate of permeability glycoprotein (P-gp), the pharmacokinetics (PK) and pharmacodynamics (PD) of LPV/RTV may be affected by the magnitude of P-gp expression, an efflux transporter protein encoded by the *ABCB1* gene [7]. P-gp may be highly expressed in some patients which may lead to poor absorption of LPV/RTV therapy as evidenced in HIV-1 infected children [8]. It is therefore predicted that similar PK effects associated with variability of P-gp expression may also occur in patients infected with SARS-CoV-2. Poor absorption of LPV/RTV may consequently lead to therapeutic failure of this antiviral therapy meaning patients are vulnerable to severe adverse clinical outcomes or even death. The effects of LPV/RTV associated with the *ABCB1* genetic variability has been investigated in HIV but there was no evidence for such associations in SARS-CoV-2 due to a lack of studies assessing this effect in SARS-CoV-2 infection. However, it is reasonably assumed that such genetic effects of *ABCB1* may also exist for SARS-CoV-2 infection causing COVID-19. Although metabolism of LPV/RTV may also be predominantly affected by CYP450 enzymes of CYP3A4/5 and organic anion transporter protein 1B1 (OATP1B1); however, due to avoiding genetic complexity, the present study will only consider genetic polymorphisms of *ABCB1* affecting safety or efficacy of LPV/RTV.

There are approximately 50 SNPs of the *ABCB1* gene as reported elsewhere [9]; therefore, it is extremely difficult to predict which SNP would be linked to PK effects of LPV/RTV affecting efficacy or safety in COVID-19 patients. However, from the findings of previous studies, it can be inferred that during this emergency situation researchers need to focus on the *ABCB1* C3435T genetic polymorphism and should assess the efficacy or safety end points since it is the most prevalent and mostly studied SNP and has some correlation with LPV/RTV treatment in HIV patients [10]. Although the correlation was with a different virus, it should be noted here that pharmacogenomics of *ABCB1* gene was affecting host PK effects of LPV/RTV, not the pathogenic virus, hence the type of virus was involved may not be the most important factor. Instead, there is a need to focus on the efficacy or safety assessment of LPV/RTV associated with *ABCB1* C3435T genetic polymorphism for COVID-19 patients infected with SARS-CoV-2. How much of the world population that may be affected by carrying this genetic polymorphism can be gauged from *ABCB1* C3435T SNP frequency in different ethnic groups as obtained from the literature review.

## Prevalence of the *ABCB1* C3435T genetic polymorphism in world population

Prevalence of *ABCB1* C3435T genetic polymorphism in different ethnic groups was obtained from the literature review. Phenotypes were predictively assigned based on carrying characteristic alleles. For example, patients carrying two copies of the rs1045642/C3435T SNP of the *ABCB1* gene were predicted to be high expressors of P-gp and were potentially considered as risk phenotypes. In a comparative fashion, participants carrying one copy of the rs1045642/C3435T SNP of *ABCB1* were predicted to be low expressors of P-gp and were also potentially considered as risk phenotypes. However, participants carrying no mutation of the rs1045642/C3435T SNP of *ABCB1* were predicted to be normal expressors of P-gp and were potentially considered as no-risk/normal phenotypes.

From the literature review, it was demonstrated that risk phenotypes carrying either one or two copies of the rs1045642/C3435T SNP of the *ABCB1* gene were highly prevalent in Europe (76.8%; 95% CI: 75–78) [11–17], followed by America (67%; 95% CI: 65–69) [18–23], Asia (63.5%; 95% CI: 62–65) [24–30] and Africa (41.4%; 95% CI: 37–46) [31,32], respectively as shown in Table 1. This might indicate that COVID-19 patients from Europe, Asia and America may be more vulnerable to either therapeutic failure or toxicity of LPV/RTV if *ABCB1* C3435T genetic variability is not considered. Patients from European countries were predictively identified as being as highest risk of either LPV/RTV treatment failure or toxicity associated with *ABCB1* C3435T genetic polymorphism, suggesting this should be considered in future clinical studies.

**Table 1.** Prevalence of genotypes associated with *ABCB1* C3435T genetic polymorphism in different population.

Study (year)	Country	Continent	Sample size	Genotype frequency	
				CC (%)	CT/TT (%)
Mahdieh *et al.* (2018)	Iran	Asia	388	38.9	61.1
Park *et al.* (2015)	South Korea	Asia	2188	43.1	56.9
Tang *et al.* (2012)	China	Asia	670	35.8	64.2
Lakhan *et al.* (2009)	India	Asia	325	14.5	85.5
Vahab *et al.* (2009)	India	Asia	242	2.9	97.1
Kim *et al.* (2006)	South Korea	Asia	160	41.9	58.1
Seo *et al.* (2006)	Japan	Asia	210	33.3	66.7
Anselmi *et al.* (2013)	Italy	Europe	1324	27.9	72.1
Sałagacka *et al.* (2011)	Poland	Europe	292	25.0	75.0
Szoeke *et al.* (2009)	Scotland	Europe	285	18.9	81.1
Ufer *et al.* (2009)	Germany	Europe	221	23.1	76.9
Shahwan *et al.* (2007)	Ireland	Europe	366	15.6	84.4
Sills *et al.* (2005)	Scotland	Europe	400	18.2	81.8
Siddiqui *et al.* (2003)	UK	Europe	515	21.4	78.6
Tavares *et al.* (2018)	Brazil	America	309	31.7	68.3
Calderón-Cruz *et al.* (2015)	Mexico	America	276	30.1	69.9
Santos *et al.* (2011)	Brazil	America	1212	35.4	64.6
Benish *et al.* (2010)	USA	America	356	39.3	60.7
Krupoves *et al.* (2009)	Canada	America	606	22.6	77.4
Estrela *et al.* (2008)	Brazil	America	320	40.6	59.4
Tazzite *et al.* (2016)	Morocco	Africa	128	45.3	54.7
Ameyaw *et al.* (2000)	Kenya	Africa	80	70.0	30.0
Ameyaw *et al.* (2000)	Sudan	Africa	51	52.0	48.0
Ameyaw *et al.* (2000)	Ghana	Africa	206	67.0	33.0

## Hypothesis

**Figure 1.**
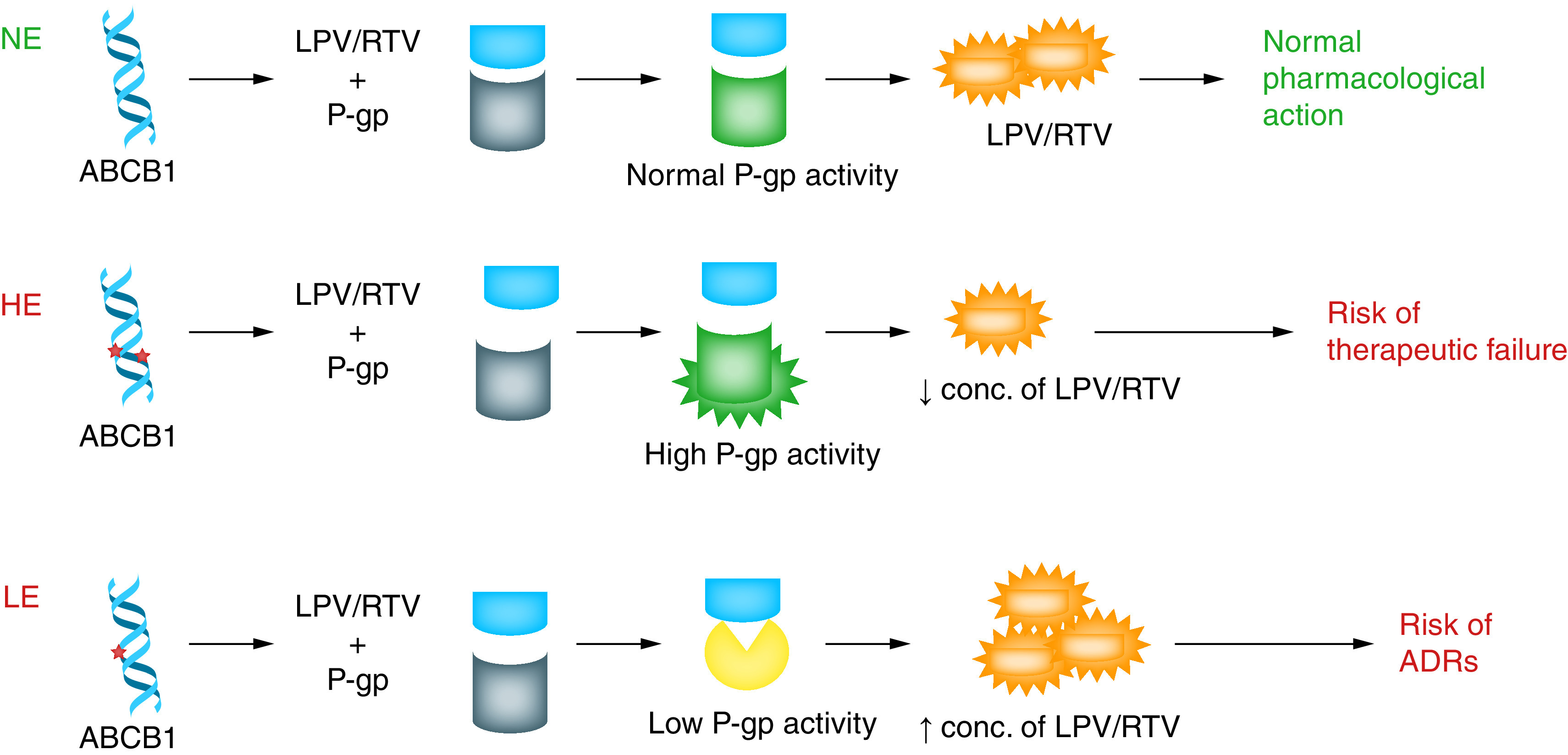
Predictive effects of the *ABCB1* C3435T mutation on the efficacy or safety of lopinavir & ritonavir. ADR: Adverse drug reaction; HE: High expressor; LE: Low expressor; LPV: Lopinavir; NE: Normal expressor; P-gp: Permeability glycoprotein; RTV: Ritonavir.

It is worth mentioning here that the association of the *ABCB1* C3435T genetic polymorphism with increased risk of major adverse cardiovascular events of clopidogrel (prodrug) in coronary artery disease patients has already been well established [33–35]. From collating overall evidence for the association of *ABCB1* C3435T genetic polymorphism with therapeutic failure of LPV/RTV in HIV infection of AIDS patients and in cardiovascular disease with another class of medication (e.g., clopidogrel), it is therefore hypothesized that “COVID-19 patients treated with LPV/RTV inheriting *ABCB1* pharmacogene in general and in particular *ABCB1* C3435T genetic polymorphism might be predisposed to either therapeutic failure or toxicity of this antiviral therapy in considerable proportion of patients” as shown graphically in Figure 1.

## Future implications of hypothesis

Amid this emergency situation, it is suggested to genotype the *ABCB1* C3435T genetic polymorphism in order to make LPV/RTV therapy more precise to overcome adverse clinical events or deaths associated with COVID-19. Every life is important in respect to his/her family, society and even for the respective country. Implementation of this ‘hypothesis’ may expected to improve clinical outcomes of many COVID-19 patients taking LPV/RTV from all over the world.

Countries like USA, UK, Australia, etc., where precision medicine initiatives are in clinical operation for many therapeutic classes of drugs or the countries having the infrastructure facilities to screen the *ABCB1* C3435T genotype testing, it is suggested to start genetic testing of this variant for patients with COVID-19 and provide personalized antiviral therapy accordingly. This can be implemented by testing the *ABCB1* C3435T polymorphism with the following groups of patients with COVID-19. Group-1 patients will be severely critically ill patients or patients who have already died. Group-2 patients will be those who are in improving clinical conditions. Group-3 will be those patients who had complete recovery from COVID-19. All of these groups of patients must be taking LPV/RTV. The clinical outcomes of these groups of patients inheriting *ABCB1* C3435T genetic polymorphisms may thus correlate the associations between these by adjusting with all other covariates.

If it appears that efficacy of LPV/RTV were significantly associated with this SNP of *ABCB1* in COVID-19 patients, then this genetic approach may be applied to other SNPs of *ABCB1* as well as genetic polymorphisms of CYP450 gene of *CYP3A4/5* affecting safety or efficacy of LPV/RTV in patients with COVID-19. For example, a study conducted by Bellusci *et al.* showed that the *ABCB1* C1236T SNP reduces the absorption of LPV/RTV leading to therapeutic failure of this antiviral therapy in HIV-1 infected children [8], which may also be applicable to patients with COVID-19. It is pertinent to mention here that metabolism of LPV/RTV may also be affected by CYP3A4 and the genetic association of this metabolic enzyme encoded by the *CYP3A4* gene has been shown in numerous studies with HIV patients indicating that certain selective SNP of *CYP3A4* may significantly lower the clearance of LPV/RTV and may produce drug toxicity [36–38]. The findings and observations of these studies may also be applicable for COVID-19 patients taking LPV/RTV affecting drug safety and should also be considerable genetic factors in future clinical studies. Since LPV/RTV is also a substrate of OATP1B1 encoded by the *SLCO1B1*, therefore, this transporter gene should also consider in future clinical studies to optimize efficacy or safety of these antiviral drugs.

Future studies should also consider cost–effectiveness of pharmacogenomic testing adoption in routine clinical practice. Although the cost–effectiveness of different pharmacogenomic testing have been investigated in several continents, predominantly Europe and North America where the estimated costs of pharmacogenomic testing in different countries were considerably different; however, most of these studies found pharmacogenomic testing to be cost-effective and recommended that it should be integrated into routine clinical practice [39–42].

It is also notable that besides considering genetic effects, future studies should also consider drug–drug interactions (DDIs) of LPV/RTV for optimizing safety and efficacy. This is because a clinical trial of LPV/RTV conducted by Cao *et al.* in adults hospitalized patients with severe COVID-19 reported that gastrointestinal adverse events were more common in LPV/RTV treatment group compared with standard care, whilst other serious adverse effects with LPV/RTV treatment group were respiratory failure, severe anemia, shock, acute kidney injury etc. These adverse clinical effects consequently leading to the early cessation of LPV/RTV treatment in COVID-19 patients [5]. Although this trial did not report whether these adverse drug reactions were due to DDIs of LPV/RTV, however, it is likely that such adverse events might occur in COVID-19 patients due to taking multiple medications leading to DDIs. A meta-analysis conducted by Alhumaid *et al.* assessing the safety and efficacy of LPV/RTV in COVID-19 patients concluded that a greater number of adverse events were reported for LPV/RTV treatment group compared with other antivirals or no antiviral treatments group [43].

Furthermore, Baralic *et al.* revealed that inflammation, cardiotoxicity and dyslipidemias were the main risks of LPV/RTV treatment in COVID-19 patients. Also, it has been suggested that since LPV/RTV may increase the expression of multiple genes involved in immune response and lipid metabolism, this drug combination should be used with caution in SARS-CoV-2-infected patients with cardiovascular diseases, autoimmune diseases, or acquired and hereditary lipid disorders [44]. From the perspectives of overall current findings, it may be concluded that a holistic approach is needed that will consider genetic effects, drug–drug interactions as well as comorbidities to optimize safety and efficacy of LPV/RTV in patients with COVID-19.

## Conclusion

Considerable proportions of the world population were predictively identified as being at risk of either therapeutic failure or toxicity of LPV/RTV due to them carrying the *ABCB1* C3435T genetic polymorphism. Since no selective antiviral treatment for COVID-19 has been developed yet, although a high priority is given from the perspectives of world scientists, it is hypothesized that COVID-19 patients treated with LPV/RTV inheriting *ABCB1* pharmacogene in general and in particular the *ABCB1* C3435T genetic polymorphism might be predisposed to either therapeutic failure or toxicity of this antiviral therapy in a considerable proportion of patients.

## Future perspective

Association of *ABCB1* genetic polymorphisms with the efficacy or safety of LPV/RTV in SARS-CoV-2 infection is unknown due to not taking COVID-19 patients into account. The hypothesis presented here may open a new window for the assessment of efficacy or safety of LPV/RTV in COVID-19 patients in future clinical studies by considering genetic variability of the *ABCB1* gene as well as other genetic factors affecting PK/PD effects of these antiviral drugs. These genetic considerations may facilitate precision medicine of LPV/RTV in viral infections.

**Executive summary**

- Lopinavir (LPV) and ritonavir (RTV) are substrates of permeability glycoprotein encoded by the gene *ABCB1*.
- Risk phenotypes associated with carrying the *ABCB1* C3435T genetic polymorphism were considerably prevalent in the world population of different ethnic groups and may affect the efficacy or safety of LPV/RTV.
- Future clinical studies are warranted to implement genotype testing of the *ABCB1* C3435T genetic mutation in patients with coronavirus disease-2019 taking LPV/RTV in order to optimize efficacy or safety of these antiviral drugs.
